# Supplementary material for: Edge time series components of functional connectivity and cognitive function in Alzheimer’s disease
Source: Brain Imaging Behav. Author manuscript; Available in PMC 2025 Feb 1. (PMC10844434; doi:10.1007/s11682-023-00822-1)
Supplement: supplementary [file NIHMS1950641-supplement-supplementary.docx]

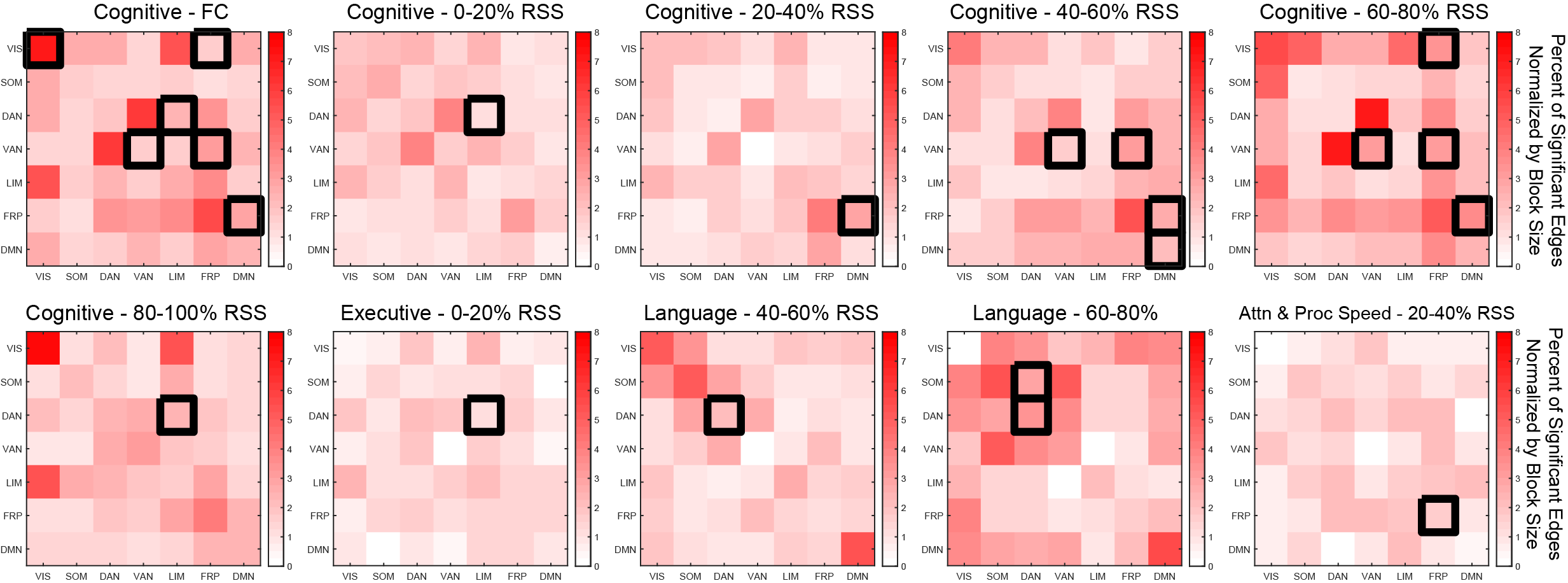


**Supplementary Figure 1. Significant blocks as assessed with the neuropsychological domain score scrambling null model.**

**
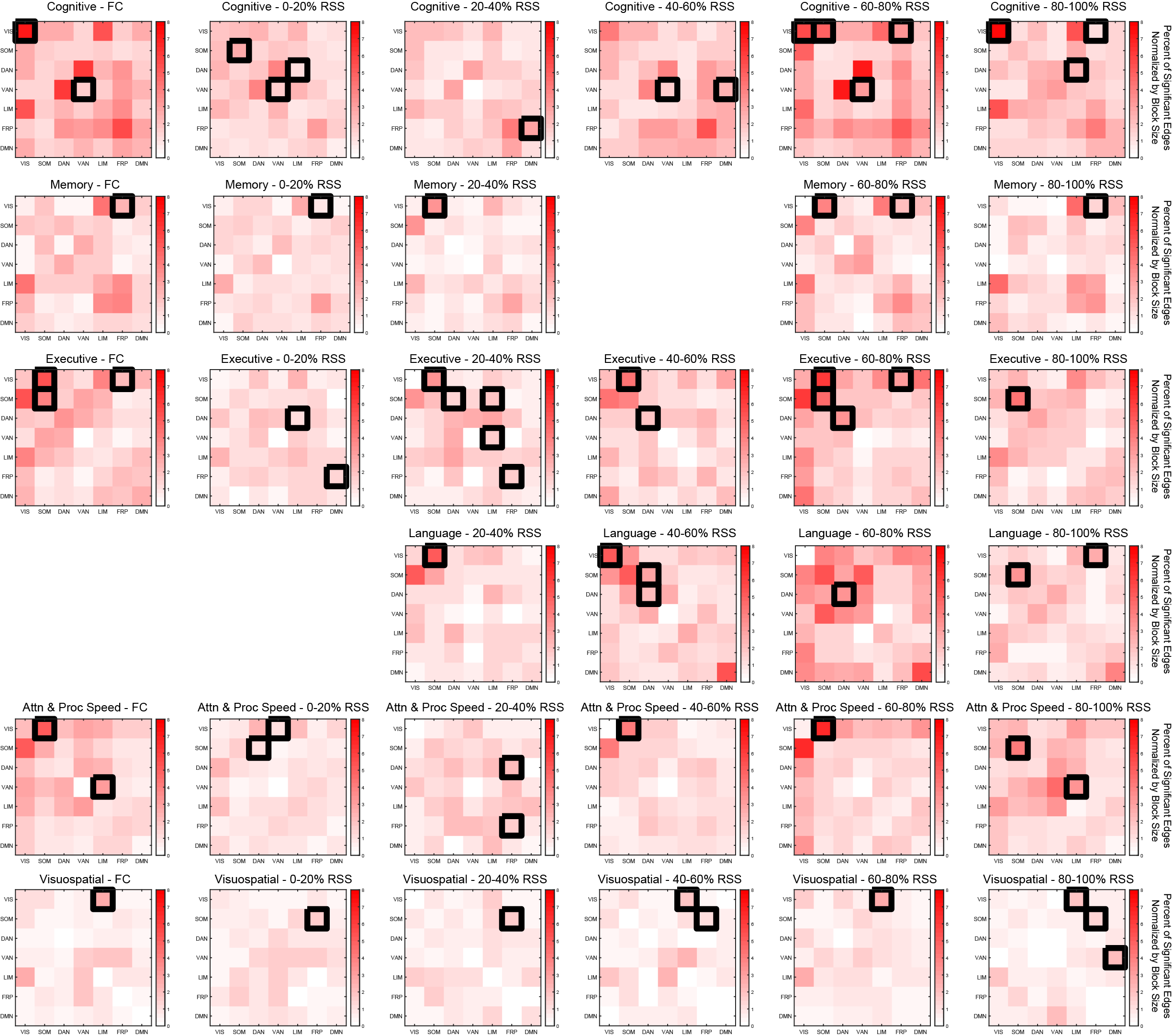
**

**Supplementary Figure 2. Significant blocks as assessed with the resting state network block structure scrambling null model.**

**
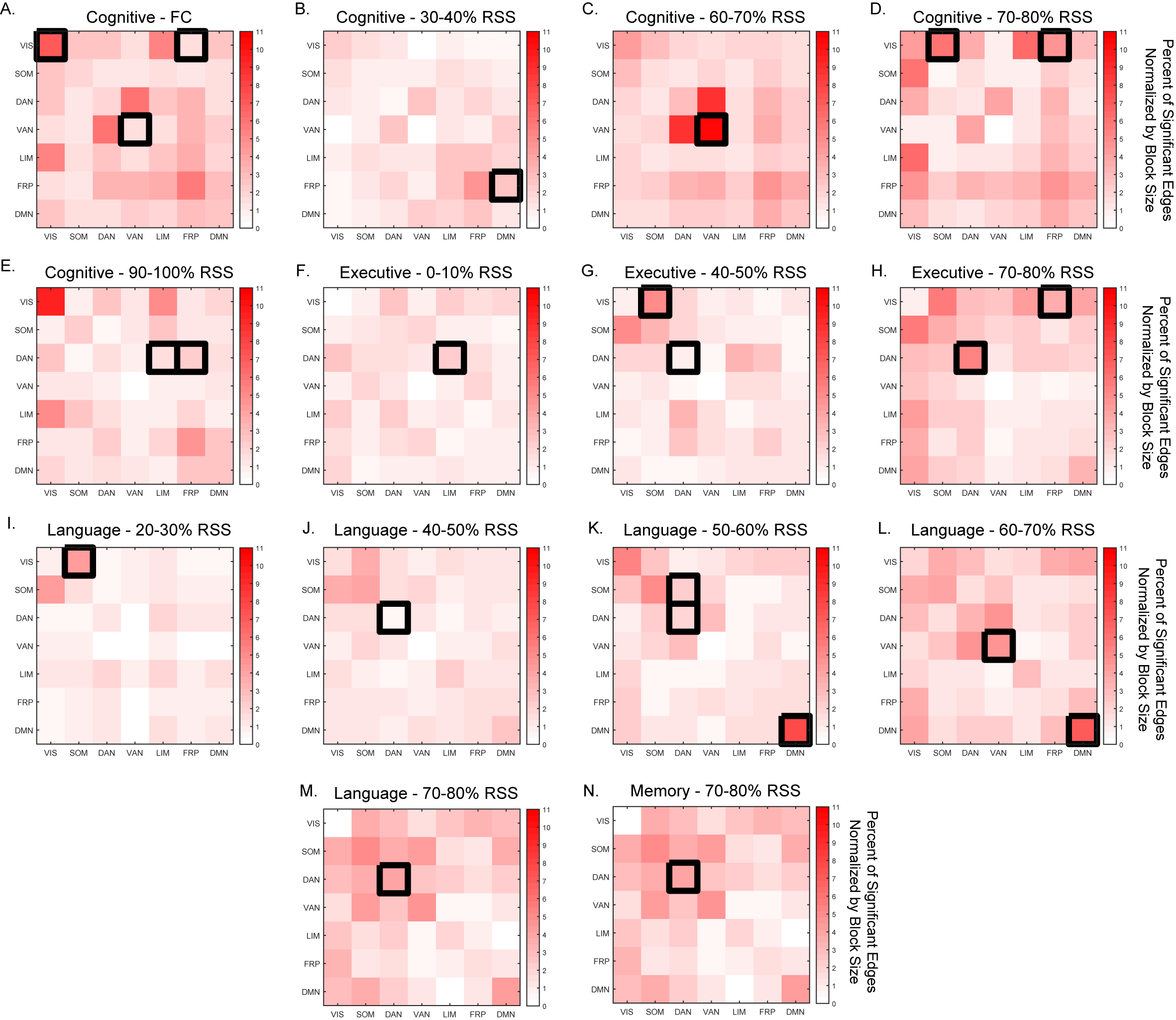
**

**Supplementary Figure 3. Significant blocks assessed from a decile split into ten FCcs. Only blocks that were significant in both null models are reported here.**

**
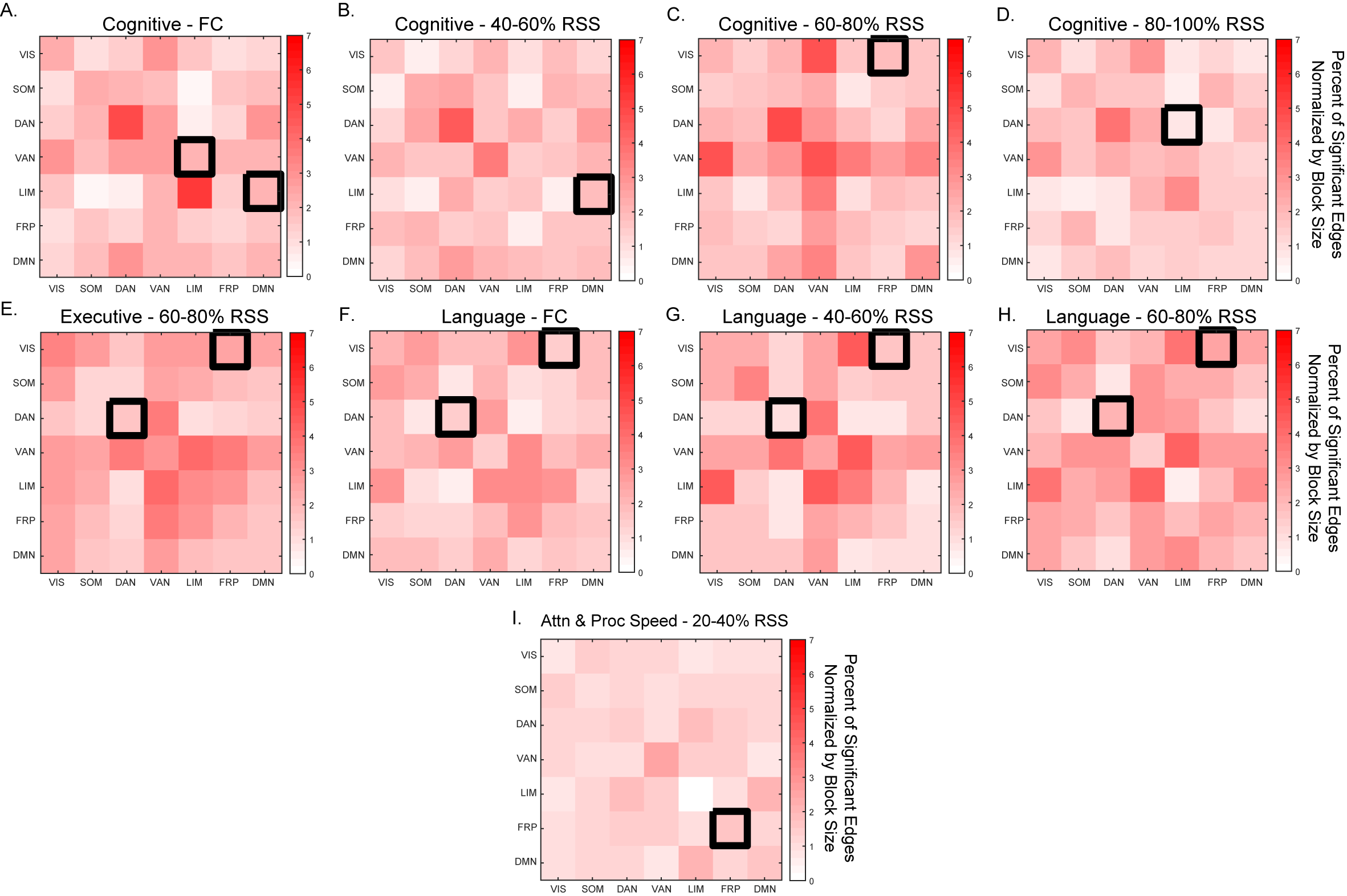
Supplementary Figure 4. Significant blocks assessed form a Schaefer 300 node parcellation data split into 5 bin FCcs. Only blocks that were significant in both null models are reported here.**

**
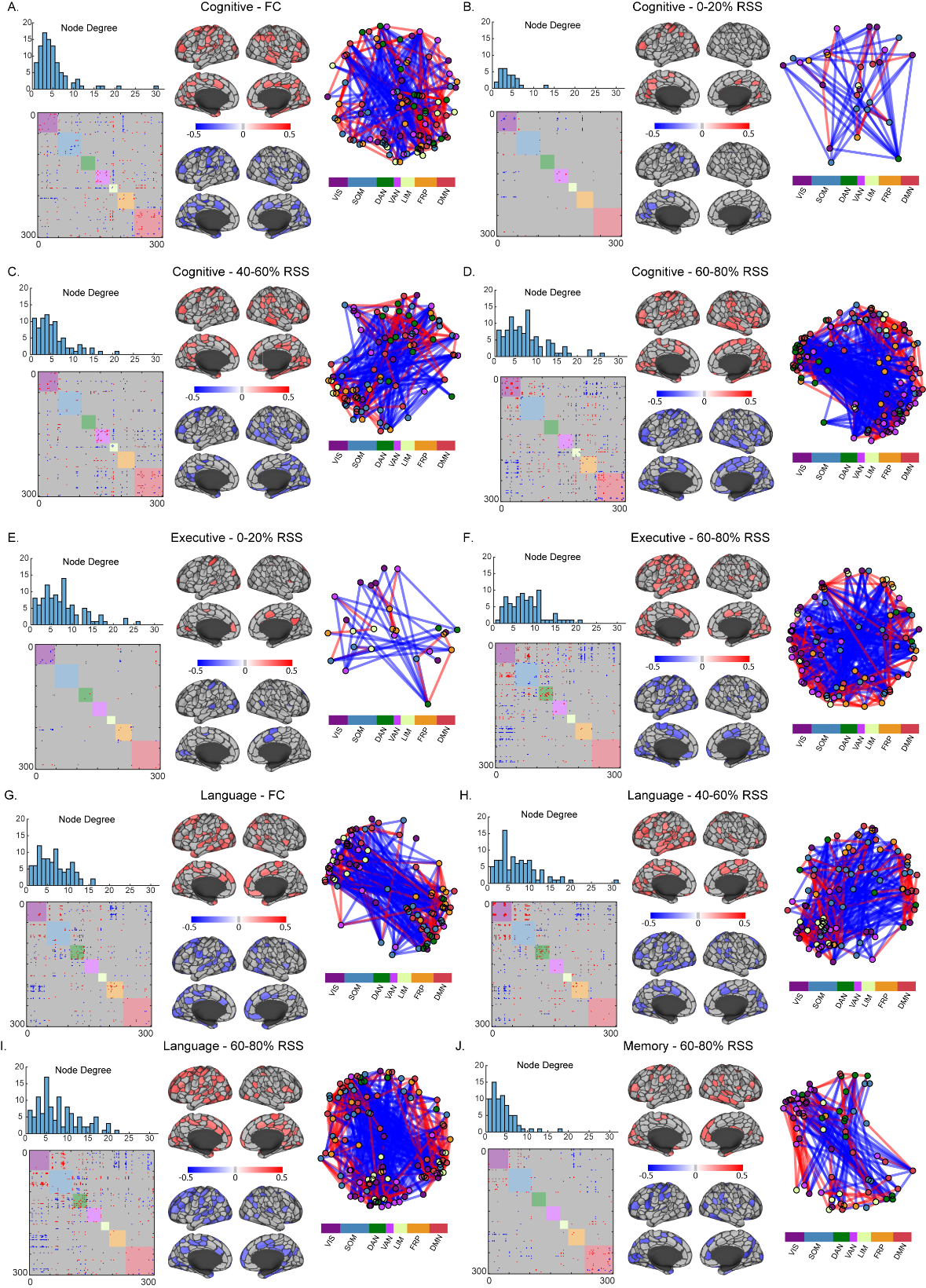
**

**Supplementary Figure 5. Block-fee Network-Based Statistics significant FC component-neuropsychological domain correlations from the Schaefer 300 node parcellated data and five RSS binned FC components.**
